# HLA haplotypes associated with hemochromatosis mutations in the Spanish population

**DOI:** 10.1186/1471-2350-5-25

**Published:** 2004-10-21

**Authors:** Arantza Pacho, Esther Mancebo, Manuel J del Rey, Maria J Castro, Desamparados Oliver, Miguel García-Berciano, Luis González, Pablo Morales

**Affiliations:** 1 Immunology. Hospital Universitario "12 de Octubre". Carretera de Andalucia. 28041. Madrid, Spain

## Abstract

**Background:**

The present study is an analysis of the frequencies of HLA-A and -B antigens and HLA haplotypes in two groups of individuals homozygous for the two main *HFE *mutations (C282Y and H63D) and a group heterozygous for the S65C mutation.

**Methods:**

The study population includes: 1123 healthy individuals, 100 homozygous for the C282Y mutation, 138 homozygous for the H63D mutation and 17 heterozygous for the S65C mutation. *HFE *and HLA alleles were detected using DNA-based and microlymphocytotoxicity techniques respectively.

**Results:**

An expected significant association between C282Y and the HLA-A3/B7 haplotype was found, but other HLA haplotypes carrying the -A3 antigen were found: HLA-A3/B62 and HLA-A3/B44. Also, a significant association between H63D mutation and HLA-A29/B44 haplotype was found, and again other HLA haplotypes carrying the HLA-A29 antigen were also found: HLA-A29/B14 and HLA-A29/B62. In addition, the S65C mutation seems to be associated with a HLA haplotype carrying the HLA-A26 antigen.

**Conclusion:**

These findings clearly suggest that HLA-A3/B7 and HLA-A29/B44 are the ancestral haplotypes from which the C282Y and H63D mutations originated, respectively. The frequencies of these mutations in different populations, their geographical distribution, and the degree of the statistical association to the ancestral haplotypes, suggest that the H63D mutation must have occurred earlier than the C282Y mutation.

## Background

Hereditary hemochromatosis (HH) is an autosomal recessive disease common in northern European populations. HH is characterized by increased iron absorption and deposition in the liver, pancreas, heart, joints and pituitary gland. Without treatment, death may occur from cirrhosis, primary liver cancer, diabetes or cardiomyopathy. In 1996, the HH gene (*HFE*) was cloned and located on the short arm of chromosome 6 (6p21.3) [1], 4 megabases (Mb) telomeric to the major histocompatibility complex (MHC). A single point mutation 845 G>A (exon 4), changing cysteine at position 282 to tyrosine (C282Y) is identified in 85 to 100% of patients with HH in populations of northern European descent, but it is found in only 60% of cases from Mediterranean populations [2]. Two other mutations, 187 C>G (exon 2), a histidine to aspartate change at amino acid 63 (H63D) and 193 A>T (exon 2), a serine to cysteine change at amino acid 65 (S65C) appear to be associated with milder forms of HH and may increase risk of disease in persons heterozygous for C282Y mutation [3,4].

C282Y lies within a Celtic ancestral haplotype which includes the human MHC (HLA) haplotype HLA-A3/B7 [5]. The HLA-A3/B7 haplotype was reported in HH patients in many European and populations of European descent [5-8] but HLA-A3/B14, HLA-A3/B35 and others were also reported [9-11]. The predominance of the HLA-A3 associated haplotypes on hemochromatosis chromosomes, and the pattern of their distribution in the world, led Simon et al [5] to propose the founder hypothesis, postulating that the hemochromatosis mutation was a rare event that occurred once on a particular chromosome which was subsequently modified by recombinations involving both HLA-B and HLA-A alleles and population migrations, producing the varied haplotype associations that were described.

In contrast, the H63D substitution is not restricted to European populations: allele frequencies greater than 5% are found in countries bordering the Mediterranean, in the Middle East, and in the Indian subcontinent. H63D seems to be associated with HLA-A29 and HLA-B44 [3,12].

A few studies have been performed on the distribution of the S65C mutation in Europe and other countries. In 1999, Barton et al [13] identified the S65C mutation in Alabama hemochromatosis probands and found that this mutation was linked to a haplotype characterized by HLA-A32; and recently, Couto et al [14] found that this mutation is in linkage disequilibrium with the HLA-A29/B44 haplotype.

The aim of this study is to find the HLA antigens and haplotypes associated with the three main mutations in the *HFE *gene in a sample of the Spanish population.

## Methods

### Individuals

A total of 100 unrelated individuals homozygous for the C282Y mutation, 138 unrelated individuals homozygous for the H63D mutation and 17 individuals heterozygous for the S65C mutation were selected for this study. These were subjects in whom *HFE *genotyping has been previously performed on a medical care basis because of a presumptive diagnosis of hemochromatosis. In addition, 1113 unrelated, apparently healthy subjects were used as controls for the study. In addition, HLA typing was performed in 230 individuals whom *HFE *genotyping was negative for the three mutations.

### HLA-A and -B typing

HLA-class I typing was performed on freshly collected venous blood samples by the standard complement-dependent microlymphocytotoxicity assay using commercially available alloantisera.

### DNA extraction and HFE amplification

Genomic DNA from whole blood samples was extracted by standard protocols. Polymerase chain reaction (PCR) with the pair of primers HEMEx2-5' (5'-CTT TGG GCT ACG TGG ATG ACC) and HEMEx2-3' (5'-CTG GCT TGA AAT TCT ACT GGA AAC C) was used to amplify exon 2 of the *HFE *gene. To amplify exon 4, a second set of oligonucleotides was used: HEMEx4-5' (5'-GGT GTC GGG CCT TGA ACT ACT ACC) and HEMEx4-3' (5'-A CAT ACC CCA GAT CAC AAT GAG G).

The following conditions were used for the PCR reactions: five minutes denaturation at 94°C, 40 cycles of 15 seconds denaturation at 95°C, 15 seconds annealing at 57°C and 30 seconds extension at 72°C. PCR products coming from exons 2 and 4 were 101 and 228 base pairs (bp), respectively.

### Digestion with mutation-specific restriction endonuclease

Following the PCR amplifications, aliquots (17 μl) of the reaction mixture were digested with the restriction endonucleases *Bcl *I (exon 2), *Hinf *I (exon 2) and *Rsa *I (exon 4) for 3 hours following the protocol recommended by the manufacturer (Promega, Madison, WI). The H63D mutation destroys the *Bcl *I site in the 101 bp PCR product, so while normal DNA is cut into two fragments of 38 and 63 bp, the mutated DNA is not cut. The S65C mutation destroys the *Hinf *I site in the 101 bp PCR product, so while normal DNA is cut into two fragments of 47 and 54 bp, the mutated DNA is not cut. The C282Y mutation creates a new *Rsa *I site, the 228 bp DNA product digested with this enzyme is cut into two fragments of 145 and 83 bp in the normal allele, while in the mutated DNA three fragments of 145, 29 and 54 bp are generated after digestion. The digested products were size resolved in 10% acrylamide gel and detected by staining with ethidium bromide.

### Statistical analysis

Allele and haplotype frequencies were estimated using Arlequin V2.0 software [15]. The haplotype frequencies were computed using the Expectation-Maximization algorithm [16]; this procedure is an interactive process aimed at obtaining maximum-likelihood estimates of haplotype frequencies from multi-locus genotype data when the gametic phase is unknown. The existence of association between *HFE *mutations and HLA-A and -B alleles and haplotypes was calculated by 2 × 2 comparison tables and p values were corrected according to the number of alleles or haplotypes compared [17] and using Yates corrected Chi^2 ^and Fisher's tests. Odds ratios were calculated as previously described [18].

## Results

The allele frequencies of the HLA-A and -B antigens found in the C282Y carriers group, in comparison with the frequencies in the control population are listed in Table 1. As expected, significant associations were found for HLA-A3 and -B7, but HLA-B62 also shows significant association. The association of HLA-A3 is stronger than that of HLA-B7 or -B62. The frequencies of the HLA-A/B haplotypes in the homozygous group in comparison with the haplotype frequencies in the control population are listed in Table 2. Three haplotypes are significantly associated with the C282Y mutation: HLA-A3/B7, HLA-A3/B62 and HLA-A3/B44.

**Table 1:** Allele frequencies of HLA-A and HLA-B antigens in the C282Y homozygous group in comparison with the control group.

	C282Y HOMOZYGOTES			CONTROLS		C282Y HOMOZYGOTES			CONTROLS
	
HLA	Freq (n = 200)	OR	p	Freq (n = 2226)	HLA	Freq (n = 200)	OR	p	Freq (n = 2226)
A1	0.070	0.69	N.S.	0.097	B8	0.035	0.81	N.S.	0.044
**A3**	**0.425**	**6.23**	**<10^-7^**	**0.106**	**B7**	**0.260**	**3.67**	**<10^-7^**	**0.089**
					**B62**	**0.075**	**2.74**	**0.030**	**0.028**
A29	0.035	0.54	N.S.	0.062	B44	0.170	1.19	N.S.	0.146
A30	0.010	0.16	0.093	0.060	B18	0.015	0.27	0.120	0.088
A2	0.195	0.67	N.S.	0.264	B35	0.095	0.77	N.S.	0.119
A11	0.060	0.75	N.S.	0.078	B51	0.055	0.60	N.S.	0.088
A26	0.020	0.42	N.S.	0.046	B49	0.025	0.67	N.S.	0.036
A28	0.025	0.59	N.S.	0.041	B60	0.015	0.42	N.S.	0.035
A32	0.020	0.57	N.S.	0.034	B14	0.010	0.28	N.S.	0.035
A23	0.030	0.95	N.S.	0.031	B38	0.010	0.33	N.S.	0.029
A33	0.010	0.36	N.S.	0.027	B27	0.025	0.88	N.S.	0.028
A25	0.020	0.97	N.S.	0.020	B50	0.005	0.21	N.S.	0.023
A31	0.025	1.56	N.S.	0.016	B65	0.015	0.64	N.S.	0.023

Freq: Frequency n: Total number of alleles in each group OR: Odds ratio p: Significance level N.S.: Not significant

**Table 2:** Frequencies of HLA-A/B haplotypes in the two groups homozygous for H63D and C282Y in comparison with the control group.

HAPLOTYPE	H63D HAPLOTYPES Freq (n = 276)	p	OR	C282Y HAPLOTYPES Freq (n = 200)	p	OR	CONTROLS Freq (n = 2226)
A1/B8	0.02513	N.S.		0.01500	N.S.		0.0246
A2/B7	0.03612	N.S.		0.02859	N.S.		0.0239
A2/B44	0.08254	N.S.		0.04037	N.S.		0.0443
A2/B51	0.04839	N.S.		0.00000			0.0404
A2/B35	0.01545	N.S.		0.03543	N.S.		0.0258
A3/B14	0.00000			0.00000			0.0005
**A3/B7**	0.00725	N.S.		**0.20275**	**<10^-7^**	**7.29**	**0.0343**
**A3/B62**	0.00000			**0.03379**	**10^-5^**	**16.11**	**0.0024**
**A3/B44**	0.00409	N.S.		**0.08989**	**<10^-^**	**12.13**	**0.0079**
A11/B27	0.01812	N.S.		0.00000			0.0064
A11/B35	0.00362	N.S.		0.00862	N.S.		0.0204
A24/B35	0.00794	N.S.		0.01478	N.S.		0.0233
**A29/B44**	**0.13261**	**<10^-7^**	**3.93**	0.02999	N.S.		**0.0378**
**A29/B14**	**0.01449**	**<0.01**	**32.7**	0.00000			**0.0005**
**A29/B62**	**0.01591**	**<0.01**	**32.7**	0.00000			**0.0005**
A30/B18	0.01087	N.S.		0.00000			0.0278
A33/B14	0.00000			0.00500	N.S.		0.0121

Freq: Frequency n: Total number of haplotypes in each group OR: Odds ratio p: Significance level N.S.: Not significant

The allele frequencies of the HLA-A and -B antigens in the H63D homozygous group in comparison with the frequencies in the control population are listed in Table 3. Significant associations were found for HLA-A29 and -B44. Again, the association of the HLA-A antigen (HLA-A29) is stronger than HLA-B44. The frequencies of the HLA-A/B haplotypes are also listed in Table 2, and three HLA-A/B haplotypes (with the same HLA-A antigen, HLA-A29) are significantly associated with the H63D mutation: HLA-A29/B44, HLA-A29/B14 and HLA-A29/B62.

**Table 3:** Allele frequencies of HLA-A and HLA-B antigens in the H63D homozygous group in comparison with the control group.

	H63D HOMOZYGOTES			CONTROLS		H63D HOMOZYGOTES			CONTROLS
	
HLA	Freq (n = 276)	OR	p	Freq (n = 2226)	HLA	Freq (n = 276)	OR	p	Freq (n = 2226)
A1	0.061	0.60	N.S.	0.097	B8	0.050	1.14	N.S.	0.044
A3	0.086	0.80	N.S.	0.106	B7	0.068	0.75	N.S.	0.089
					B62	0.028	1.00	N.S.	0.028
**A29**	**0.199**	**3.65**	**<10^-7^**	**0.062**	**B44**	**0.264**	**2.10**	**2.10^-5^**	**0.146**
A30	0.036	0.58	N.S.	0.060	B18	0.086	0.98	N.S.	0.088
A2	0.286	1.12	N.S.	0.264	B35	0.076	0.60	N.S.	0.119
A11	0.036	0.44	N.S.	0.078	B51	0.097	1.12	N.S.	0.088
A26	0.029	0.61	N.S.	0.046	B49	0.032	0.89	N.S.	0.036
A28	0.021	0.52	N.S.	0.041	B60	0.011	0.30	N.S.	0.035
A32	0.039	1.16	N.S.	0.034	B14	0.014	0.40	N.S.	0.035
A23	0.047	1.52	N.S.	0.031	B38	0.032	1.10	N.S.	0.029
A33	0.007	0.26	N.S.	0.027	B27	0.036	1.29	N.S.	0.028
A25	0.032	1.60	N.S.	0.020	B50	0.018	0.76	N.S.	0.023
A31	0.011	0.67	N.S.	0.016	B65	0.039	1.74	N.S.	0.023

Freq: Frequency n: Total number of alleles in each group OR: Odds ratio p: Significance level N.S.: Not significant

From the 17 individuals heterozygous for the S65C mutation, 7 (20%) were HLA-A26 versus 4.6% found in the control population (p = 0.02, OR = 5.29). No association with HLA-B has been found.

The significant associations did not change if we used a control group of 230 individuals without *HFE *mutations.

## Discussion

Populations of homozygous individuals for C282Y and H63D are optimal groups to study the HLA haplotypes in which these mutations preferentially appear. To our knowledge, Barton [19] and the present work are the only studies of associations between *HFE *mutations and HLA antigens and haplotypes in homozygous probands. The paper by Barton and Acton [19] presents haplotype frequencies assessed by family studies where phase could be set; in our paper, the haplotype frequences are estimated because the probands and controls are unrelated individuals. The low frequency of the S65C mutation makes the sampling of homozygous probands difficult and imposes the use of heterozygous individuals for the analysis.

### C282Y and HLA

The strong association between the HLA-A3/B7 haplotype and the C282Y mutation indicates that this haplotype is the main one associated with this mutation in the Spanish population. However, other haplotypes are also associated: HLA-A3/B62 and HLA-A3/B44. This finding supports the founder hypothesis of Simon et al [5]: the ancestral haplotype where the C282Y mutation occurred on the ancestral haplotype HLA-A3/B7 and subsequent recombinations involving both HLA-B and HLA-A alleles produced the varied haplotype associations that have been found. Thus, we found many HLA-A/B haplotypes in our C282Y group, but only three HLA-A3 bearing haplotypes are statistically associated with this mutation. The two less frequent haplotypes (HLA-A3/B44 and HLA-A3/B62) have been observed in other populations in association with HH [20,21].

In addition, the high frequency of the HLA-A3/B7 haplotype makes other HLA antigens and haplotypes have reduced frequencies in respect to the controls. It is interesting to see that haplotypes with high-frequency in the Spanish population, such as HLA-A30/B18 and HLA-A2/B51 are absent in the C282Y homozygous group (Table 2). These HLA haplotypes are not *contaminated *by the C282Y mutation, and up until now, these haplotypes may be considered as *protector *haplotypes.

### H63D and HLA

Porto et al [3] and Cardoso et al [22] found individual associations between HLA-A29 and non-classical forms of iron overload in linkage disequilibrium with H63D, and a strong linkage disequilibrium between H63D and all A29 containing haplotypes assigned in a large population of normal portuguese families.

In the present work we found a strong association between the HLA-A29/B44 haplotype and the H63D mutation. Our finding agrees with the association of HLA-A29/B12(44) and hemochromatosis described in the Danish population [21].

H63D and HLA-A29-bearing haplotypes follow a pattern of associations similar to that described for C282Y and HLA-A3-bearing haplotypes. This promotes speculation that HLA-A29/B44 is the ancestral haplotype from which the H63D mutation emerged, since other HLA-A29 carrying haplotypes are also statistically associated with the mutation (HLA-A29/B14 and HLA-A29/B62, see Table 2), confirming the results reported by Cardoso et al [22] in the normal Portuguese population. Studies in other populations might lend support to whether HLA-A29/B44 is the ancestral haplotype of the H63D mutation, and HLA-A29/B14 and HLA-A29/B62 are specific Spanish haplotypes associated with H63D mutation.

### C282Y and H63D mutations: which one is older?

In an attempt to establish the relative age of C282Y and H63D mutations, we have analysed the geographical distribution, allele frequencies and HLA haplotype associations for each mutation, assuming that the mutations ocurred once and that its age is directly proportional to its geographical spread, its frequency in the population, and the number of HLA haplotypes to which they are linked. On the other hand, a strong association of a particular *HFE *mutation to a particular HLA haplotype could mean that the mutation arose more recently, since the lower number of ruptures and recombinations of the original haplotype would reflect that a shorter time has passed.

Merryweather-Clarke et al [23] analysed 2978 samples from probands distributed world-wide and showed that the C282Y mutation was most prevalent in northern European populations and absent from samples of non-European subjects (Africans, Asians, Australasians and Americans). In contrast, the H63D mutation is not restricted to European populations, being found in countries bordering the Mediterranean, in the Middle East and in the Indian subcontinent, and its allele frequency is higher than that of the C282Y mutation [23]. Our analysis of C282Y and H63D homozygous groups yielded a higher number of HLA haplotypes in association with the H63D mutation; and the frequency of HLA-A3/B7 in the C282Y homozygous group is 20%, while the frequency of HLA-A29/B44 in the H63D homozygous group is 13% (see table 2), reflecting that the HLA-A29/B44/H63D haplotype has suffered more recombinations than HLA-A3/B7/C282Y, and therefore, that HLA-A29/B44/H63D is older [24].

Altogether, these features suggest that the H63D mutation may have occurred earlier than the C282Y mutation, as has been previously proposed in studies from Italian populations [25,26].

### S65C and HLA

Few studies have been performed on the distribution, frequency and HLA association of the S65C mutation in Europe and other continents. Barton et al [13] described the linkage of the S65C mutation to a HLA-A32 haplotype in hemochromatosis probands from Alabama. Surprisingly, Couto et al [14] found the linkage of S65C (and not H63D) to the HLA-A29/B44 haplotype in a population from the Azores, although only 5 H63D homozygous and 9 S65C heterozygous individuals were studied in that work.

In the present work we find that the S65C mutation seems to be linked to HLA-A26 in the Spanish population. Further studies in other populations and with more S65C-bearing haplotypes are necessary to shed light on the generation of the S65C mutation.

## Conclusions

We have found that, in the Spanish population, the three main *HFE *mutations: C282Y, H63D and S65C, are in linkage disequilibrium with HLA haplotypes carrying the HLA-A3, -A29 and -A26 alleles, respectively. In addition, the ancestral HLA haplotypes from which C282Y and H63D mutations were originated are HLA-A3/B7 and HLA-A29/B44, respectively, and H63D is older than C282Y. Further studies in other populations using homozygous individuals for *HFE *mutations will help to identify the associated ancestral and specific haplotypes.

## Competing interests

The author(s) declare that they have no competing interests.

## Authors' contributions

Authors AP and PM conceived the study, contributed to proband characterisation, performed statistical comparisons and edited the manuscript. Authors EM, MJR, MJC, DO, MGB and LG contributed at different times to the characterisation of probands and HLA typing.

## Pre-publication history

The pre-publication history for this paper can be accessed here:


